# An endoplasmic reticulum stress-related signature featuring ASNS for predicting prognosis and immune landscape in prostate cancer

**DOI:** 10.18632/aging.205280

**Published:** 2024-01-10

**Authors:** Zhenyu Wu, Zhenquan Wu, Jie Zeng, Yaxuan Liu, Yue Wang, Huixin Li, Taolin Xia, Weitao Liu, Zhe Lin, Wenfeng Xu

**Affiliations:** 1 Department of Urology, The First People’s Hospital of Foshan, Foshan, P.R. China; 2 Department of Thoracic Surgery, Guangzhou First People’s Hospital, South China University of Technology, Guangzhou, P.R. China; 3 Department of Blood Transfusion, Shenzhen Hospital Affiliated to Southern Medical University, Shenzhen, P.R. China; 4 The First Clinical Medical College, Guangdong Medical University, Zhanjiang, P.R. China

**Keywords:** ASNS, ERS, gene signature, prostate cancer, TCGA

## Abstract

Prostate cancer (PRAD) is one of the common malignant tumors of the urinary system. In order to predict the treatment results for PRAD patients, this study proposes to develop a risk profile based on endoplasmic reticulum stress (ERS). Based on the Memorial Sloan-Kettering Cancer Center (MSKCC) cohort and the Gene Expression Omnibus database (GSE70769), we verified the predictive signature. Using a random survival forest analysis, prognostically significant ERS-related genes were found. An ERS-related risk score (ERscore) was created using multivariable Cox analysis. In addition, the biological functions, genetic mutations and immune landscape related to ERscore are also studied to reveal the underlying mechanisms related to ERS in PRAD. We further explored the ERscore-related mechanisms by profiling a single-cell RNA sequencing (scRNA-seq) dataset (GSE137829) and explored the oncogenic role of ASNS in PRAD through *in vitro* experiments. The risk signature composed of eight ERS-related genes constructed in this study is an independent prognostic factor and validated in the MSKCC and GSE70769 data sets. The scRNA-seq data additionally revealed that several carcinogenic pathways were noticeably overactivated in the group with high ERS scores. As one of the prognostic genes, ASNS will significantly inhibit the proliferation, migration and invasion abilities of PRAD cells after its expression is interfered with. In conclusion, this study developed a novel risk-specific ERS-based clinical treatment strategy for patients with PRAD.

## INTRODUCTION

Prostate cancer (PRAD) has the second highest incidence among all male malignant tumors [1]. According to estimates, there will be 34,500 PRAD deaths and roughly 268,490 new cases of PRAD in the United States by 2022. 1,414,259 new cases and 375,304 deaths were recorded worldwide in 2020 [2]. The standard treatment for localized PRAD embraces radical prostatectomy and radical radiation therapy. Although the majority of PRAD patients are cured, approximately 35% of patients with radical prostatectomy [3] and 30–50% of patients with radical radiotherapy [4] experience biochemical recurrence (BCR) within 10 years. These patients will ultimately develop castration-resistant PRAD [5], leading to death in 32–45% of patients within 15 years [6]. In order to anticipate recurrence risk and adjust active surveillance, it is essential to ascertain early biomarkers of PRAD progression and BCR [7]. Nevertheless, emerging pathological parameters have been distinguished for the early prognosis of PRAD, which still have some limitations in clinical practice [8]. Consequently, recognizing prognostic biomarkers for PRAD progression is momentous to foresee the risk of recurrence.

The principal location for protein folding and calcium storage is the endoplasmic reticulum (ER), which also regulates the formation of lipid membranes and intracellular cholesterol [9]. Contrarily, endoplasmic reticulum stress (ERS) is justified by interfering with the ER’s mechanism for folding proteins in the face of stressful conditions such as hypoxia, oxidative stress, aberrant glycosylation, and calcium homeostasis, which leads to the buildup of misfolded or unfolded proteins [10]. In addition, genetic alterations can also promote ERS and continuously activate unfolded protein response (UPR) pathways [11]. It was detected that excessive activation of ERS will deplete ATP in cells, causing autophagy and even apoptosis [12]. Furthermore, ERS plays an integral role in tumor development. It modifies the balance between tumor cell death, dormancy, and aggressive development in addition to specifically affecting how sensitive solid tumors are to chemotherapeutic treatments [13]. On the other hand, it has been shown that this situation alters the activity of immune systems in the tumor microenvironment (TME), which inhibits the growth and recurrence of cancer [14]. Accumulating researches demonstrated that ERS was associated with tumor development, aggressiveness, and response to analogous treatments in hepatocellular carcinoma [15] and breast cancer [16]. Furthermore, a recent study has displayed that the forecast of prognosis of bladder cancer patients was involved with ERS-related lncRNAs [17]. The potential function of ERS-related genes in PRAD has not yet been clarified.

In our study, we obtained transcriptome data and related clinical information based on The Cancer Genome Atlas (TCGA) database and extracted ERS-related gene sets from the Molecular Signature Database (MSigDB). Additionally, we obtained data from the Memorial Sloan-Kettering Cancer Center (MSKCC) and Gene Expression Omnibus (GEO) database. Then, we used the MSKCC and GSE70769 cohorts as the validation set and the TCGA cohort as the training set. We concentrated on researching the effect of ERS-related genes on predicting BCR risk in PRAD patients and examining their possible processes on tumor development and progression by connecting pertinent genes to PRAD clinical cases.

## METHODS

### Data collection and preprocessing

We downloaded RNA profile information in TPM format of 501 PRAD tumors and 52 normal tissues and corresponding clinical data from the TCGA database https://portal.gdc.cancer.gov/. We downloaded RNA data and related clinical data of 231 PRAD patients from the MSKCC database http://cbio.mskcc.org/cancergenomics/prostate/ [18]. In addition, we downloaded GSE70769 containing RNA expression data and corresponding clinical information of 94 PRAD patients from the GEO database https://www.ncbi.nlm.nih.gov/geo/ [19]. Next, we used the datasets IMvigor210 and GSE91016 to predict the efficiency of immunotherapy [20, 21]. We collected a single-cell RNA sequencing (scRNA-seq) dataset (GSE137829) from 6 PRAD patients and used the “seurat” R package for quality control, cell clustering, and annotation.

### Consensus clustering analysis

A total of 252 ERS-related genes were downloaded from MSigDB https://www.gsea-msigdb.org/gsea/msigdb/index.jsp and provided in Supplementary Table 1. PRAD samples were clustered into subgroups based on these genes by the Non-negative Matrix Factorization (NMF) approach with the “NMF” R package. We used the Kaplan-Meier survival curves to compare the subgroups’ biochemical recurrence-free (BCRF) survival between the subgroups. Two gene sets (c2.cp.kegg.v7.1.symbols.gmt and c7.all.v7.5.1.symbols.gmt) from the MsigDB database were extracted to estimate the differences in biological functions and immune activities between the subgroups using the Gene Set Variation Analysis (GSVA) with the “GSVA” R package. The statistically significant cutoff for GSVA was *p*.adjust < 0.05.

### Generation of ERS-related signature

We used the TCGA cohort as a training set to create ERS-related risk signatures, and used MSKCC and GSE70769 data sets to validate the performance of the risk signatures. By using univariate Cox analysis and random survival forest (RSF) analysis, we reduced the number of prognostic genes. The best ERS-associated risk signature was then created using multivariable Cox regression analysis based on the respective coefficients (β) and gene expression levels (Exp). Based on the median ERscore, we then split the patient population into high- and low-risk groups. The prognostic differences between the two patient groups were assessed using the Kaplan-Meier method. We also looked at the relationships between ERscore and age, PSA, TN stage, Gleason score (GS), and BCR among other clinical parameters. To assess the predictive importance of ERscore, both univariate and multivariate Cox analyses were performed. We simultaneously gathered the MSKCC and GSE70769 validation sets to confirm the ERscore’s predictive ability.

### Functional enrichment analysis

We performed a differential analysis between high-risk and low-risk groups. We analyzed differential genes between high and low risk groups by performing Gene Ontology (GO) enrichment and Kyoto Encyclopedia of Genes and Genomes (KEGG) enrichment. We used genomic variation analysis (GSVA) to compare the oncogenic signature pathways (h.all.v7.1.symbols) recorded in the MSigDB database between the two cohorts and screen out signature pathways with significant differences (*p*.adjust < 0.01). We used gene set enrichment analysis (GSEA) to analyze the same signature pathways and compare (FDR < 0.25, NES > 1 and *p*.adjust < 0.05). The predictive relevance of GSVA and GSEA overlapping feature paths was evaluated using the Kaplan-Meier method.

### Mutation analysis

The TCGA database was used to retrieve somatic mutations in PRAD patients. In different risk groups, the “maftools” R program may investigate particular somatic mutational variations. Next, we investigated the enrichment of oncogenes, known oncogenic pathways, co-occurring or exclusive mutations between the two groups. It was determined whether the tumor mutation burden (TMB), which represents the overall mutation count for each PRAD patient, correlated with ERscore.

### Assessing the immune landscape and tumor treatment response

Between high risk and low risk groups, we examined variations in immune cell abundance, immunological function, and immune checkpoints. Based on RNA expression patterns in PRAD patients, the Tumor Immune Dysfunction and Elimination (TIDE) algorithm http://tide.dfci.harvard.edu/ [22] was utilized to predict an immunotherapy response. ERscore and prospective immunotherapy effectiveness were correlated using the IMvigor210 and GSE91061 datasets, respectively. The “pRRophetic” R package predicted the IC50 value of the chemotherapy medication for each patient at the same time as we looked into how the two groups responded to chemotherapy.

### scRNA-seq data analysis

Next, we employed the GSE137829 dataset to investigate the single-cell characteristics of PRAD. After sample preprocessing, the “harmony” and “copykat” R packages were utilized to adjust the batch effect of samples and identify malignant cells. Utilizing the “AUCell” R package, we determined the activity of genesets associated with ERscore in various types of single cells. We divided all of the cells into high and low groups based on the AUC score. The “CellChat” R program was used to analyze variations in signaling pathways across groups with high and low ERS scores and predict cell-cell interactions.

### Cell culture and transfection

PRAD cells (PC-3 and DU145) and normal prostate epithelial cells (RWPE-1) were acquired from the American Type Culture Collection (ATCC, Manassas, VA, USA). PC-3 cells were cultured in RPMI-1640 medium; DU-145 cells were incubated in DMEM medium; RWPE-1 cells were cultured in Keratinocyte Serum Free medium. Fetal bovine serum (10% FBS) was added to all the cells, which were then grown at 37°C in a 5% CO2 environment. We applied siRNA to knock down ASNS. ASNS siRNA (sense: CCAAAUGGCAAAGUUGCAUTT, antisense: AUGCAACUUUGCCAUUUGGCT) was obtained and transfected into PC-3 cells.

### RNA extraction and qRT-PCR

Total RNA was isolated using TRIzol reagent (MRC, Cincinnati, OH, USA). Then, M-MLV Reverse Transcriptase (Promega, Madison, WI, USA) was utilized to perform reverse transcription. Subsequently, qRT-PCR was performed by the HGoTaq^®^ qPCR Master Mix (Promega, Madison, WI, USA). The following PCR primers were used: ACTB forward: 5′-CTCCATCCTGGCCTCGCTGT-3′; reverse: 5′-ACTAAGTCATAGTCCGCCTAGA-3′. ASNS forward: 5′-TGAGGAAGGCATTCAGGCT-3′; reverse: 5′-CACGCTATCTGTGTTCTTCCG-3′.

### Western blot analysis

The total protein of cells was obtained using RIPA lysis buffer (Servicebio, Wuhan, China). Equal amounts of different protein samples were separated by SDS-PAGE gel and then transferred to PVDF membrane. Membranes were blocked with 5% nonfat milk for 1 h and incubated with primary antibodies against ASNS (Biorbyt Ltd., #orb340938) or GAPDH (Aksomics, #KC-5G5) overnight at 4°C. Anti-rabbit IgG (SouthernBiotech, #4050-05) served as the secondary antibody. Blots were visualized using V370 Flatbed Photo Scanner (EPSON, Singapore).

### Cell counting Kit-8 (CCK-8) assays

Cell viability of PC-3 cells was monitored through CCK-8 (APExBio, Houston, TX, USA). PC-3 cells were seeded in the 96-well plates. The Optical Density (OD450) was evaluated on days 1–5.

### 5-Ethynyl-2′-deoxyuridine (EdU) assays

EdU assays were performed with the EdU DNA Cell Proliferation Kit (Beyotime, Shanghai, China). PC-3 cells were seeded into 96-well plates and incubated for 2 days. The EdU and the Hoechst 33342 staining were conducted based on the manufacturer’s protocol. The images were obtained using the inverted fluorescence microscope (Olympus, Singapore), and the percentage of EdU-positive cells was computed.

### Transwell assay

To evaluate the invasive ability of PRAD cells, a transwell chamber (Corning, NY, USA) was coated with Matrigel (Corning, NY, USA). The upper chambers were seeded with PC-3 cells in an FBS-free medium. Media consisting of 10% serum were added to the lower chamber. Cells were cultured at 37°C for 24 h, and the remaining cells were swept using a cotton swab. The cells that had infiltrated the chamber’s bottom were then fixed with 4% methanol at 37°C for 10 min, followed by 15 min of staining with a 0.1% crystal violet solution in the same environment. A microscope was used to count the invading cells in three randomly selected fields.

### Wound-healing assay

The wound-healing assay was performed to assess the targeted cells’ migration capacity. PC-3 cells were seeded into 6-well plates and cultured until the cells surface reached a 95% confluence. The cell surface was scratched slowly and evenly to create a blank area, and the medium was changed to an FBS-free medium. The cell migration was photographed at 0 and 24 h under the inverted microscope. The relative migration rate was calculated.

### Statistical analysis

In this work, RStudio (version 4.0.2) and GraphPad Prism 8.0 were used to perform all statistical analyses. To evaluate differences between continuous variables, the unpaired Student’s *t*-test was applied. To investigate the association between categorical parameters, the chi-square test was applied. For the majority of studies, statistical significance was experimentally fixed at a two-tailed *p* < 0.05.

### Data availability statement

The datasets in our research are publicly available, which can be found in TCGA and GEO databases.

## RESULTS

### The consensus clustering of ERS-related genes

**Figure 1:**
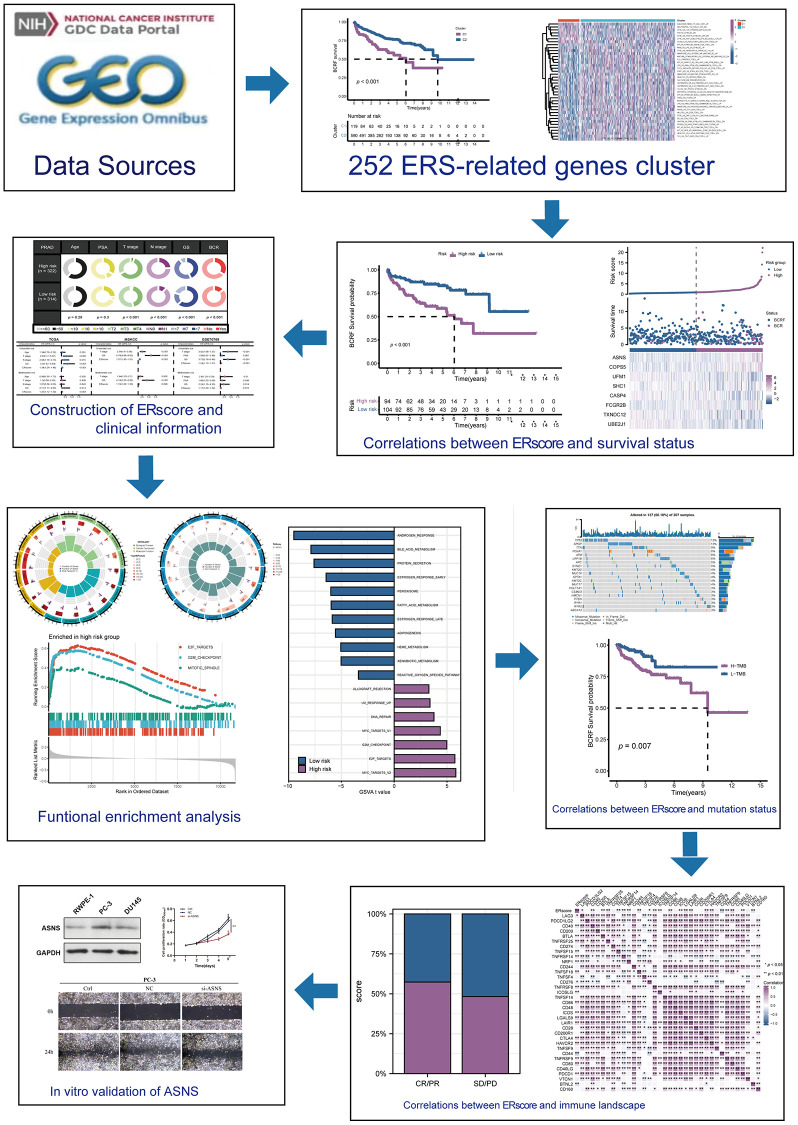
This study’s design and flowchart.

**Figure 2:**
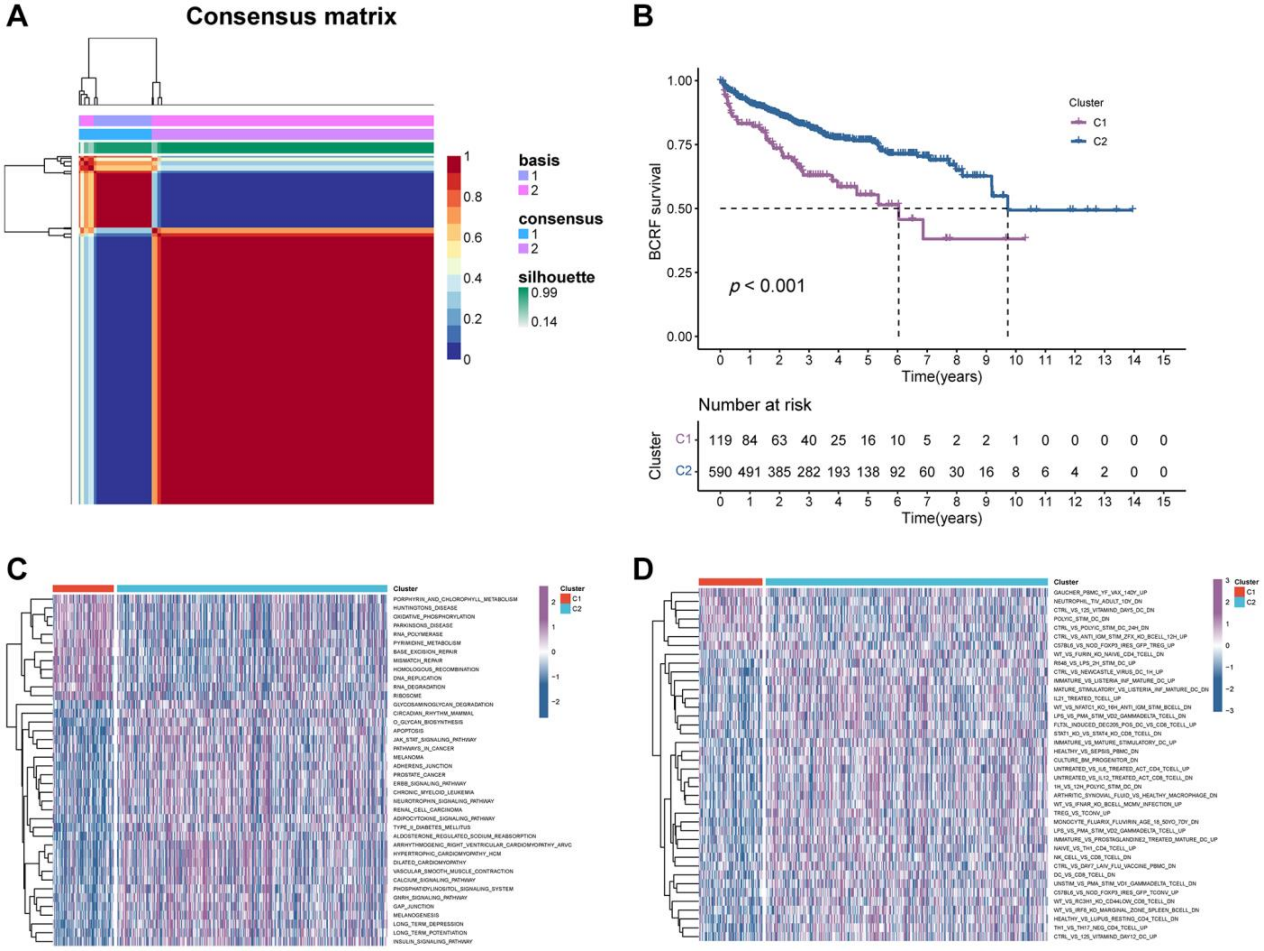
**The consensus clustering of ERS-related genes predicted the BCRF survival of PRAD patients.** (**A**) The consensus matrix (k = 2) was acquired using the NMF method. (**B**) Kaplan-Meier analysis demonstrated that cluster 2 had a significantly better BCRF survival. (**C**, **D**) GSVA analysis for pathways enrichment and immunological functions.

The flow chart of this study is shown in Figure 1. Based on the expression profile of ERS-related genes, PRAD patients were divided into two categories (Figure 2A, Supplementary Figure 1). Cluster 2 exhibited a considerably greater BCRF survival than cluster 1, according to the Kaplan-Meier analysis. (Figure 2B). GSVA enrichment analysis discovered that cluster 1 was mainly correlated with mutation-relevant pathways such as homologous recombination, base excision repair, RNA replication, and mismatch repair. On the contrary, cluster 2 was significantly enriched in glucose and amino acid metabolism including glycan biosynthesis and degradation, glycosaminoglycan degradation, propanoate metabolism, beta-alanine metabolism, and tryptophan metabolism (Figure 2C). Further results of GSVA also revealed remarkable differences in immunological functions between the two clusters (Figure 2D). Taking together, our findings uncovered that the two ERS-related subgroups were well separated and distinct in prognostic outcomes and biological functions.

### Construction of ERS-based model

**Figure 3:**
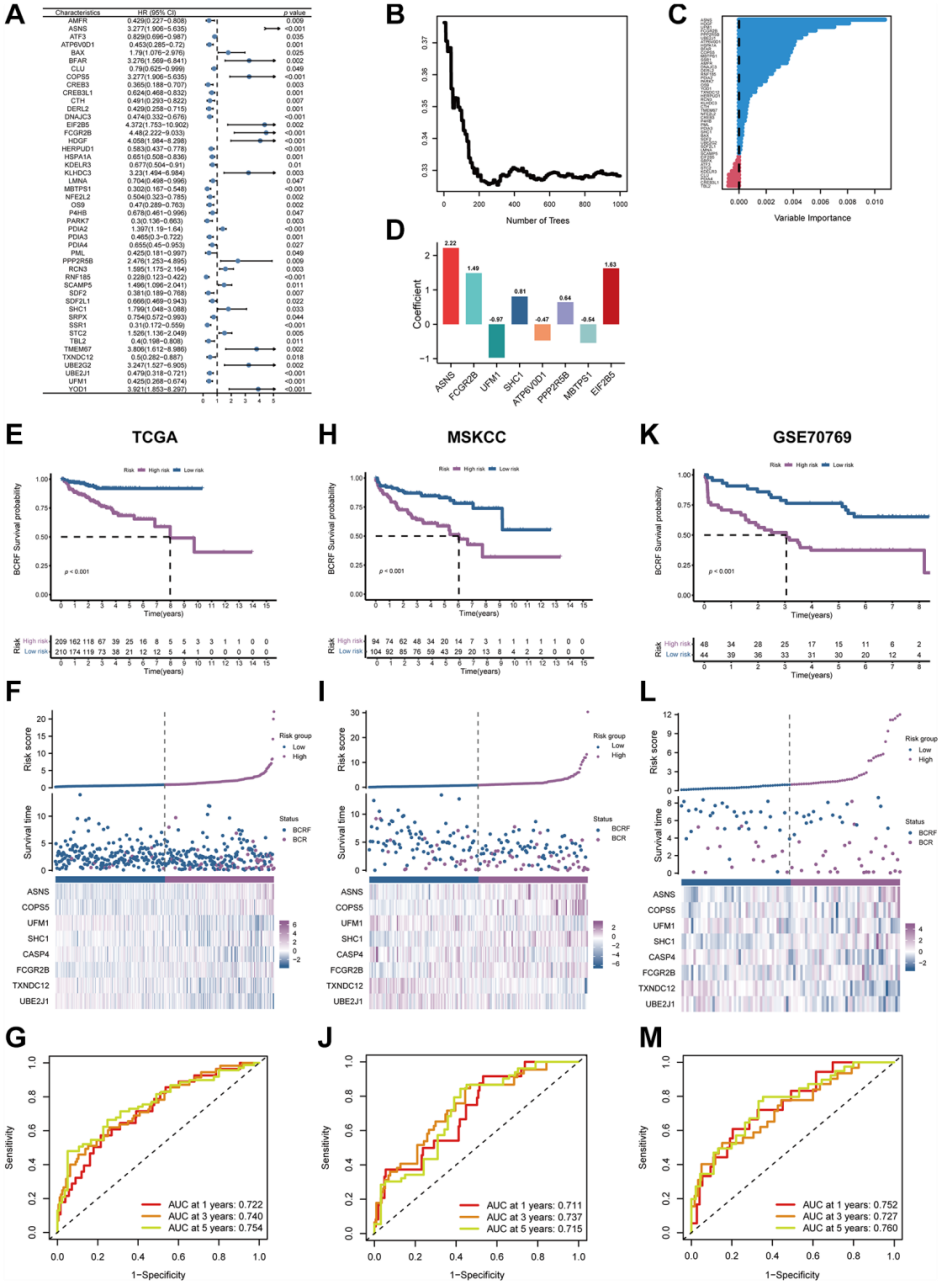
**The establishment of ERscore and verification of its prognostic efficiency.** (**A**) Univariate Cox regression analysis recognized 47 prognosis-associated genes. (**B**) Correlations between error rate and classification trees. (**C**) The relative importance of prognosis-associated genes. (**D**) The corresponding coefficients of ERscore-constructed genes. (**E**) The Kaplan-Meier method unveiled a significantly worse BCRF survival of the high-risk cohort compared to the low-risk cohort. (**F**) The illustrations of all patient’s survival condition, risk variations, and ERscore distributions. (**G**) ROC approach validated that ERscore was a promising prognostic indicator. (**H**–**M**) The outcomes of MSKCC and GSE70769 cohorts also validated ERscore’s prognostic value.

**Figure 4:**
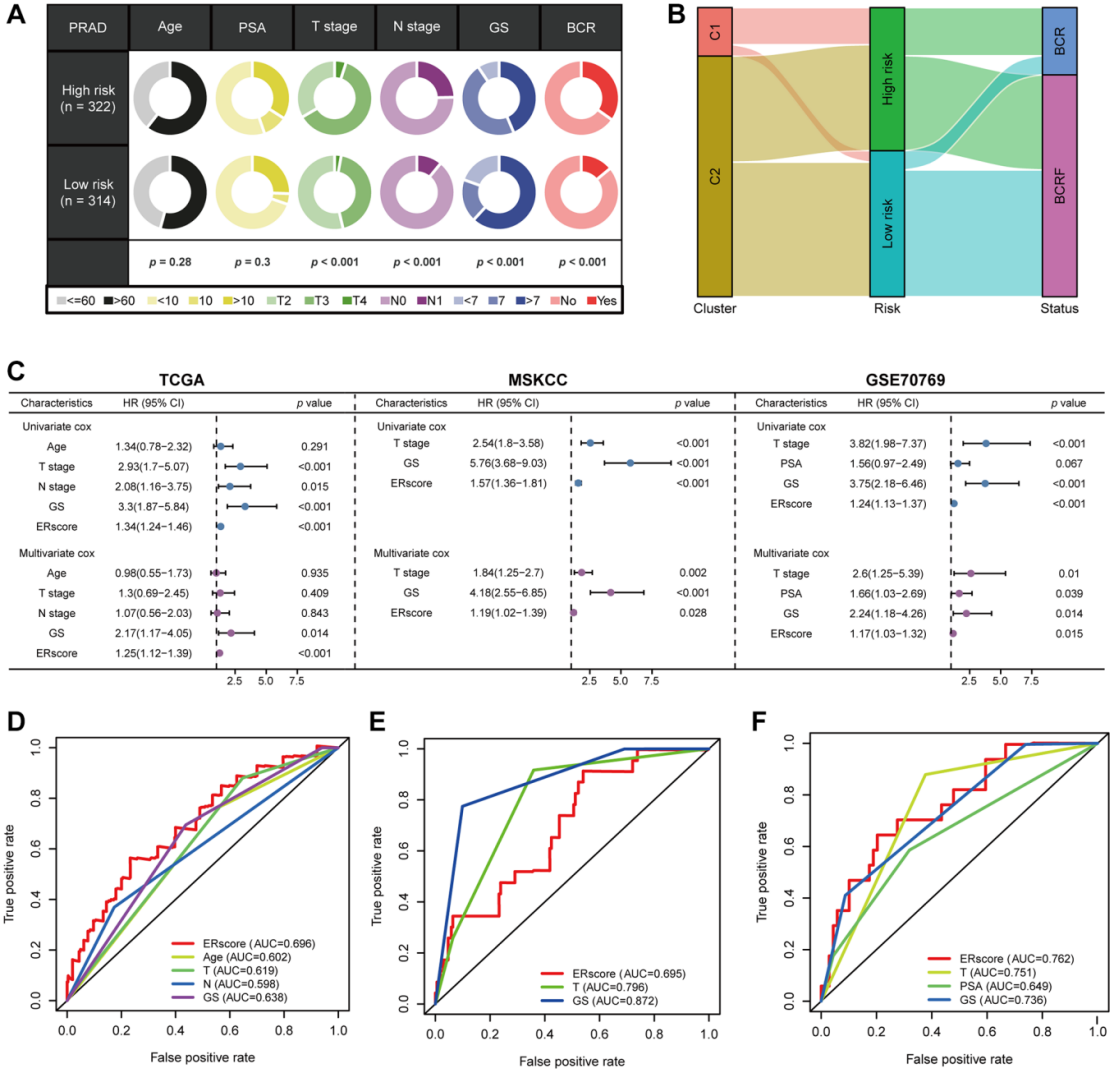
**The prognostic value of ERscore and clinical variables.** (**A**) Relationship between ERscore and clinical features. (**B**) Correlations between consensus clustering and ERscore of PRAD patients. (**C**) Univariate and multivariate Cox regression analyses of ERscore and clinical features in TCGA, MSKCC, and GSE91061 cohorts. (**D**–**F**) ROC method revealed the prognostic significance of ERscore in TCGA, MSKCC, and GSE70769 cohorts, respectively.

We firstly identified 47 genes associated with prognosis in the TCGA cohort. (Figure 3A). Fourteen candidates were further selected through RSF for model development using minimum depth techniques (Figure 3B, 3C). Using multivariate Cox regression, the last eight relevant genes were selected to create an ERscore, namely ASNS, FCGR2B, UFM1, SHC1, ATP6V0D1, PPP2R5B, MBTPS1, and EIF2B5. The formula was:


$$\text{ERscore}=\sum_{i=1}^{8}\left(Exp_{i}\times \beta_{i}\right)$$

(Table 1, Figure 3D). We classified patients into high- and low-risk groups based on the median ERscore. The high-risk group’s BCRF survival was considerably lower than that of the low-risk group’s (Figure 3E). Figure 3F displayed the ERscore distribution, survival status, and ERscore profile for these individuals. For 1-, 3- and 5-year BCRF survival, the AUCs of the ERscore were 0.722, 0.740, and 0.754, respectively (Figure 3G). We conducted further investigation and discovered that a greater ERscore was linked to a poorer TN stage, a higher GS, and a higher likelihood of BCR (Figure 4A). Moreover, high-risk patients were more likely to come from cluster 1 with a poorer prognosis (Figure 4B). Furthermore, in univariate Cox regression, it was found that ERscore and clinical traits were significantly related to BCRF survival. ERscore was revealed to be an independent predictive predictor (Figure 4C). The accuracy of ERscore’s prediction was also supported by ROC analysis (AUC = 0.696, Figure 4D). The prognostic value of ERscore was also confirmed through the validation set (MSKCC and GSE70769) (Figures 3H–3M and 4E, 4F). These results suggest that ERscore has the potential to guide clinical treatment.

**Table 1:** The prognostic significance of the 8-genes signature.

**ERS-related gene**	**Coef**
ASNS	2.220676006
FCGR2B	1.489245428
UFM1	−0.971498354
SHC1	0.808327395
ATP6V0D1	−0.469498761
PPP2R5B	0.642209931
MBTPS1	−0.541691574
EIF2B5	1.627293875

### Functional enrichment analysis

**Figure 5:**
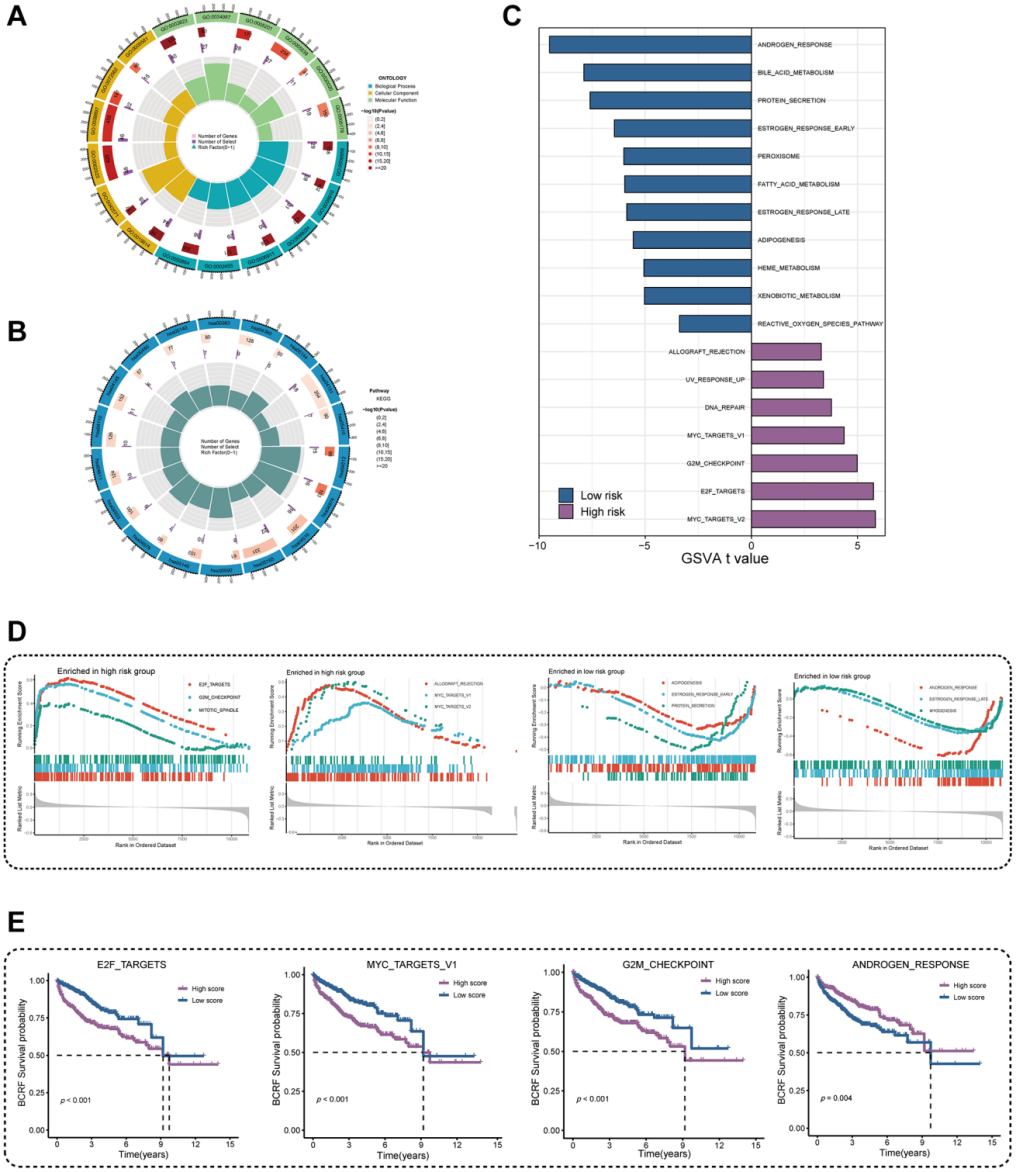
**Investigation of underlying mechanism regarding ERscore.** (**A**) GO enrichment analysis of ERscore. (**B**) KEGG pathway analysis of ERscore. (**C**) Determination of oncogenic hallmark pathways in terms of the ERscore risk cohorts utilizing GSVA. (**D**) The GSEA outcomes for the hallmark pathways between the high- and low-risk patients. (**E**) Kaplan-Meier curve uncovered the BCRF survival in overlapping hallmark pathways between GSVA and GSEA.

We performed GO and KEGG enrichment analyses in order to investigate the underlying mechanism of ERscore. The PI3K-Akt signaling pathway, the p53 signaling pathway, mitotic spindle assembly, chromosomal segregation, immunoglobulin receptor binding, and B cell receptor signaling pathways were all demonstrated to be associated with ERscore (Figure 5A, 5B). According to the above GO and KEGG items, ERscore may be related to immunological processes, tumor mutations, and carcinogenic pathways. Following the inclusion of 50 oncogenic signature pathways in GSVA, results revealed that 7 signature pathways considerably increased in high-risk patients whereas 11 pathways dramatically reduced in low-risk patients (Figure 5C). Seven genes were highly elevated in the high-risk group, according to GSEA analysis, whereas six genes were downregulated in the low-risk group (Figure 5D). Using the Kaplan-Meier approach to analyze the intersection-derived pathways, different BCRF survival probabilities for a number of well-known oncogenic pathways were found such as E2F_TARGETS, MYC_TARGETS_V1, G2M_CHECKPOINT, and ANDROGEN_RESPONSE (Figure 5E). This suggests that ERscore has a role in a variety of biological processes, particularly carcinogenic pathways in PRAD.

### Somatic mutation analysis

**Figure 6:**
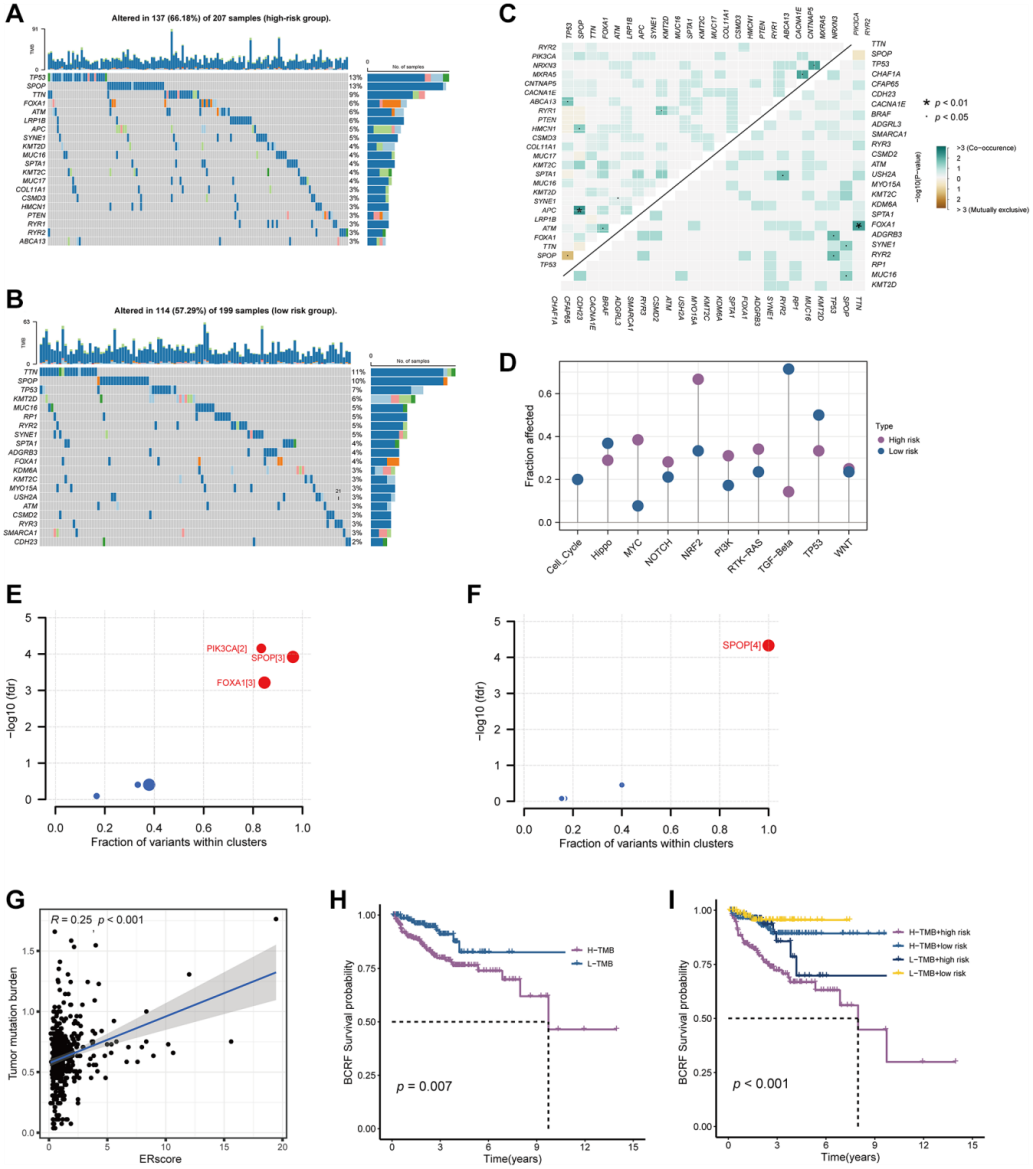
**Genetic mutations landscape in terms of the ERscore risk cohorts.** (**A**, **B**) Waterfall plots of genetic mutations in high- and low-risk groups, respectively. (**C**) The co-occurring or exclusive mutations across the top 25 mutated genes between the two cohorts. (**D**) The results of mutation enrichment of remarkable oncogenic pathways. (**E**, **F**) The investigation of tumorigenic genes in high- and low-risk groups, respectively. (**G**) The relationship of ERscore and TMB. (**H**, **I**) Kaplan-Meier curve revealed the BCRF survival in distinct TMB and ERscore groups.

Waterfall plots were used to illustrate the genetic mutation landscape between high- and low-risk populations (Figure 6A, 6B). TP53, SPOP, TTN, FOXA1, and ATM were the top five most often mutant genes in the high-risk group, while TTN, SPOP, TP53, KMT2D, and MUC16 were the top five most frequently mutated genes in the low-risk group. Furthermore, we analyzed co-occurring or exclusive mutations in the top 25 mutated genes between the two cohorts, but no significant differences were observed (Figure 6C). The MYC, NRF, and PI3K signaling pathways were greatly enhanced while the TGF-Beta signaling pathway was dramatically decreased in the high-risk group, according to mutation enrichment of carcinogenic pathways (Figure 6D). In contrast to PIK3CA and FOXA1, which were exclusively enriched in the high-risk group, SPOP was shown to be enriched in both high- and low-risk populations in our research of carcinogenic genes (Figure 6E, 6F). This shows that PIK3CA and FOXA1 have a significant role in the development of ERS-related tumors. The relationship between ERscore and TMB is also favorable (Figure 6G, 6H). Survival analysis also showed that patients with high TMB and high risk scores were associated with poor prognosis (Figure 6I).

### Immune landscape and treatment response

**Figure 7:**
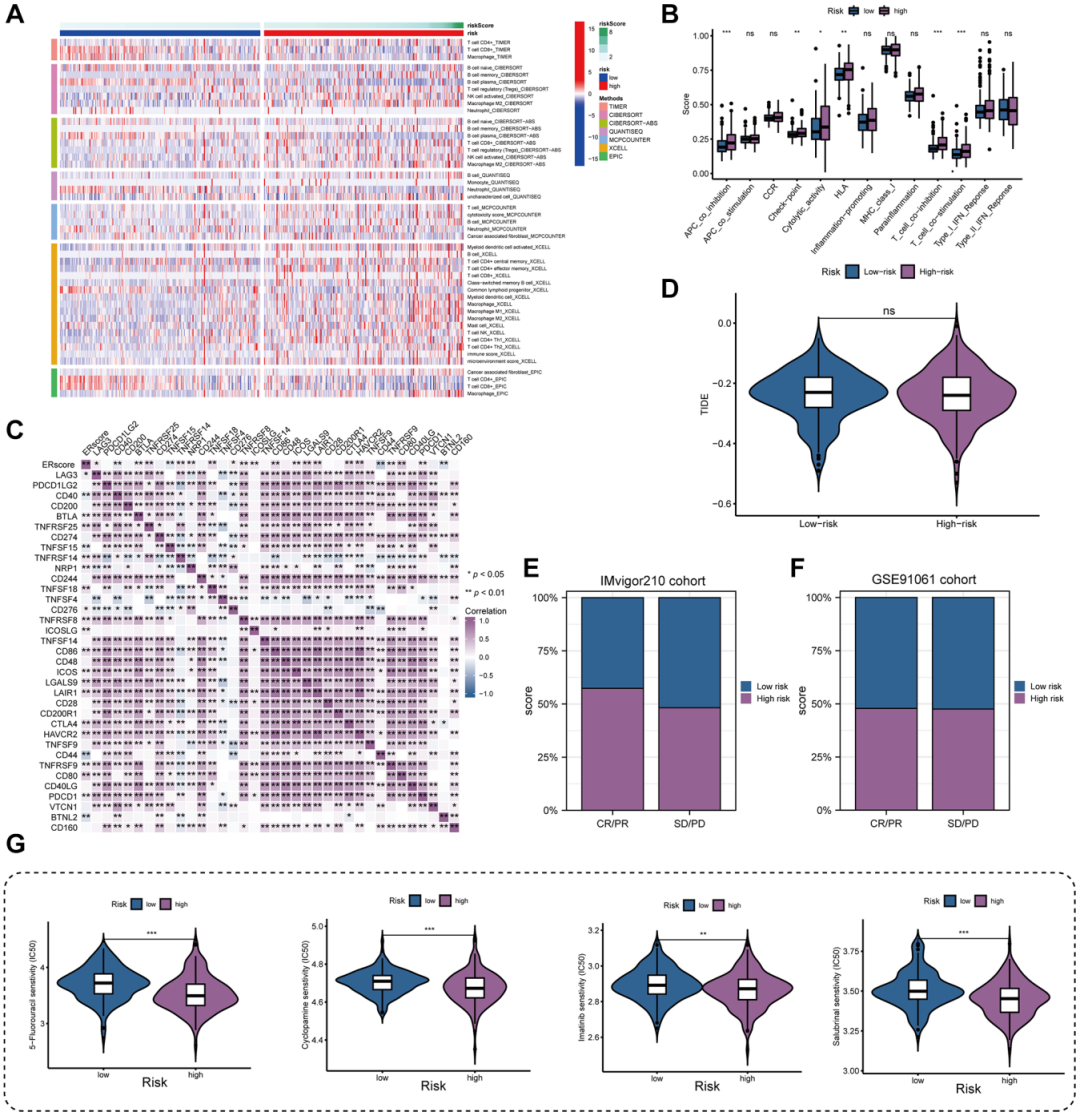
**Immune landscape and treatment response prediction.** (**A**) Estimation of immune cell infiltration in high- and low-risk teams. (**B**) Explorations of immunological responses in terms of the ERscore risk groups. (**C**) Correlations between ERscore and immune checkpoints. (**D**) TIDE algorithm identified the difference in immunotherapy response between high- and low-risk groups. (**E**, **F**) The prediction of immunotherapy response using IMvigor210 and GSE70769 cohorts. (**G**) The prediction of chemotherapy response of PRAD patients with different ERscores. ^*^p < 0.05, ^**^p < 0.01, ^***^p < 0.001. Abbreviation: ns: not significant.

By examining the immune environment of the tumor, we found that the high-risk group had more immune cells, including T cells, B cells, NK cells, and macrophages infiltrating than the low-risk group (Figure 7A). Furthermore, most immune-related pathways were significantly elevated in the high-risk group, including immune checkpoints, cytolytic activity, HLA function, and T cell costimulation (Figure 7B). In addition, we found that immunosuppressive receptor expression was higher in patients at higher risk (PD-1, CTLA4, BTLA, and LAG3) as well as immunosuppressive ligands (LGALS9 and TNFSF14) (Figure 7C). Patients in high- and low-risk groups did not significantly differ in their responses to immunotherapy, according to the TIDE algorithm (Figure 7D). The anticipated outcomes from the GSE91061 cohort did not reveal this difference, despite the fact that patients with higher ERscores in the IMvigor210 cohort had a stronger anti-PD-1 response than patients with lower ERscores (Figure 7E, 7F). We assessed the chemotherapeutic response of PRAD patients with various ERscores in considering the fact that PRAD has poor response to immunotherapy. Our results demonstrate that various chemotherapeutic agents, including 5-fluorouracil, cyclopamine, imatinib, and salubrinal, have much lower IC50 values in high-risk individuals (Figure 7G).

### scRNA-seq data analysis

**Figure 8:**
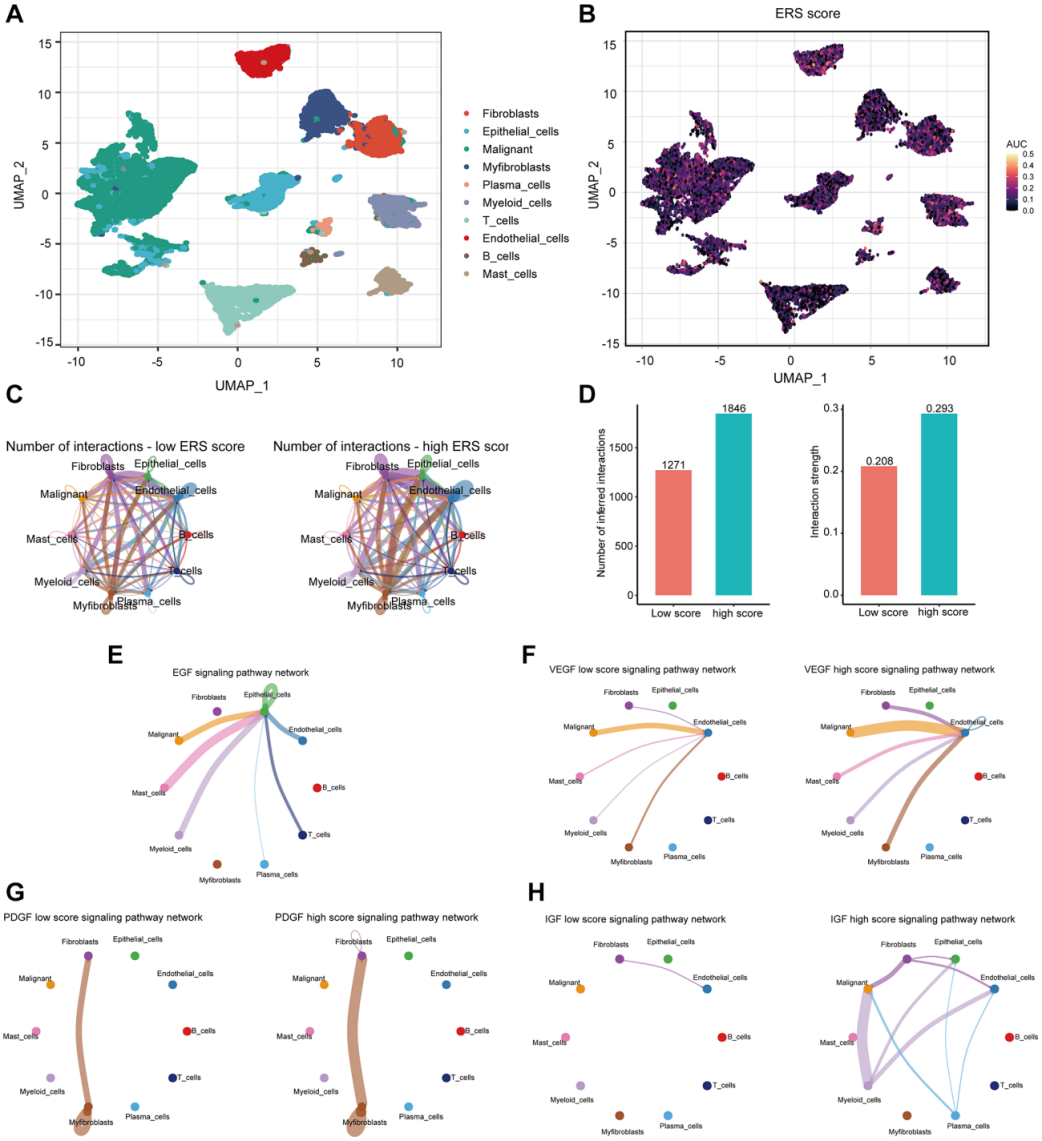
**The association of ERS-based signature with the scRNA-seq characteristics.** (**A**) UMAP plot revealed the composition of 10 main clusters derived from PRAD scRNA seq data. (**B**) The AUC score (activity) of ERS-based signature in 10 main clusters. (**C**) Circos plots showed inferred ligand-receptor interactions of the high and low ERS score groups. (**D**) Differences in intercellular interactions (number and strength) of all cells between the high and low score groups. (**E**–**H**) Circos plots displayed the differences in EGF, VEGF, PDGF, and IGF signaling pathways between the high and low score groups.

After sample preprocessing, the cells were clustered and annotated into 10 major clusters fibroblasts, epithelial cells, malignant cells, myofibroblasts, plasma cells, myeloid cells, T cells, endothelial cells, B cells, and mast cells (Figure 8A, Supplementary Figure 2). Subsequently, we divided all cells into high and low groups based on ERS-related AUC scores (Figure 8B). The high ERS score group showed enhanced intercellular interactions in number and strength based on ligand-receptor signaling (Figure 8C, 8D). Particularly, the high ERS score group exhibited elevated EGF, VEGF, PDGF, and IGF signaling pathways in comparison to the low ERS score group (Figure 8E–8H). Patients in the high ERS score group and the low ERS score group displayed different intercellular communication patterns, and the high ERS score group had considerably overexpressed carcinogenic pathways.

### *In vitro* experiments for ASNS

**Figure 9:**
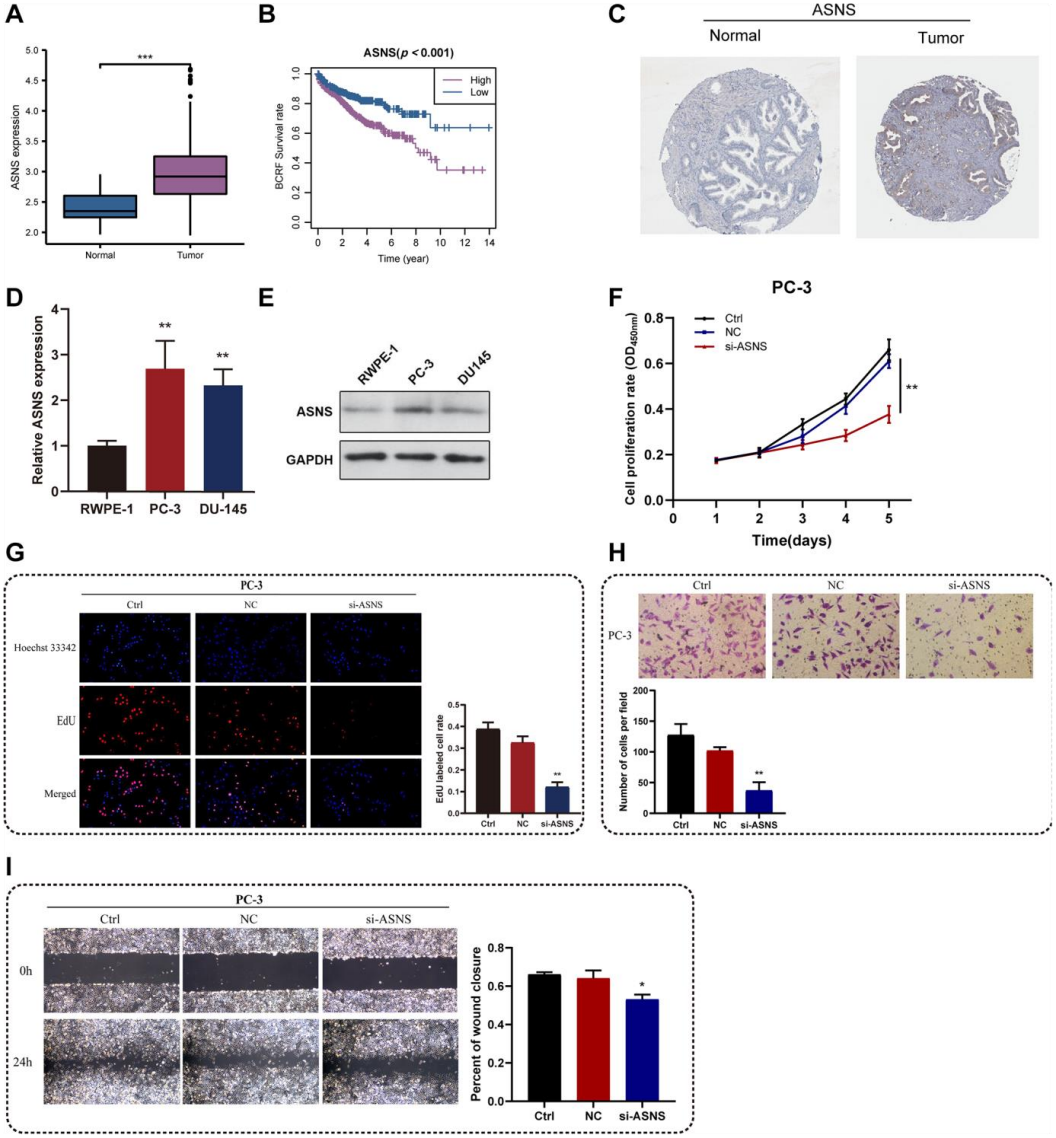
***In vitro* experiments for ASNS.** (**A**) Relative expression of ASNS in PRAD and normal tissues in TCGA cohort. (**B**) ASNS was associated with worse BCRF survival of PRAD patients in TCGA cohort. (**C**) Human Protein Atlas revealed that ASNS protein expression was relatively higher in PRAD. (**D**, **E**) The expression levels of ASNS in PC-3 and DU145 cells were relatively higher than in RWPE-1 cells using qRT-PCR and western blot. (**F**) The CCK-8 assays demonstrated the correlations between ASNS and proliferation activity. (**G**) The EdU assays showed the correlations between ASNS and proliferation activity. (**H**) The transwell assays demonstrated the correlations between ASNS and migration capacity. (**I**) The invasion assays showed the correlations between ASNS and invasive competence. ^*^p < 0.05, ^**^p < 0.01, ^***^p < 0.001. Abbreviation: ns: no significance.

Among the essential genes that make up the ERscore, ASNS was strongly associated with PRAD prognosis (Coef = 2.22). Therefore, we chose ASNS for further analysis. Firstly, TCGA investigation revealed that PRAD had much more ASNS expression than normal tissue, and that ASNS was linked to worse BCRF survival in PRAD patients (Figure 9A, 9B). Data from Human Protein Atlas https://www.proteinatlas.org/ revealed that ASNS protein expression was relatively higher in PRAD (Figure 9C). Subsequently, our *in vitro* experiments confirmed that the expression levels of ASNS in PC-3 and DU145 cells were significantly higher than those in RWPE-1 cells by qRT-PCR and Western blotting. (Figure 9D, 9E). To validate our predicted tumor-promoting role of ASNS in PRAD, we knocked down ASNS expression in PC-3 cells using siRNA and overexpressed ASNS in DU145 with the ov-ASNS vector (Supplementary Figure 3). The qRT-PCR tested the knockdown efficiency of si-ASNS, and the results prompted us to select si-ASNS sequence 2 for further experiments. Through the application of the CCK-8 and EdU assays, we discovered that suppressing ASNS expression can drastically slow PC-3 cells’ ability to divide and proliferate (Figure 9F, 9G). Additionally, we discovered that inhibiting ASNS dramatically reduced PC-3 cells’ capacity for migration and invasion using Transwell and invasion assays (Figure 9H, 9I). These findings suggest that ASNS may become a new target for patients with PRAD.

## DISCUSSION

PRAD is one of the most common malignancies of the genitourinary system and the most common cancer in men [23]. Therefore, investigating novel markers for tumor development, especially BCR, can facilitate early stratification and appropriate treatment for PRAD patients. A recent study has shown that ERS was closely linked to tumor growth and progression and may serve as a crucial target for cancer therapy [24]. It was reported that ERS can promote the apoptosis of glioma cells and may be used as a therapeutic target for glioma [25]. In addition, Yang et al. [26] recognized 8 ERS-related genes and validated the prognostic value of these genes in lung adenocarcinoma patients. In the study, we obtained ERscore by screening the ERS-related genes and confirmed that ERscore can be used as a prognostic indicator for PRAD. A previous study has demonstrated that the prognostic model constructed by miRNA can predict the prognosis of PRAD, but its predictive power (AUC = 0.711) was lower than our study (AUC = 0.754) [27]. The findings of a single database are also unconvincing, despite the fact that earlier research has identified novel traits for predicting the prognosis of PRAD patients based on the TCGA database [28, 29]. In this work, we combined numerous datasets to produce ERscore and used a variety of approaches to investigate ERscore’s biological roles. We anticipate that ERscore will aid clinical treatment plans for PRAD patients.

Furthermore, GO and KEGG analysis showed that ERscore may be closely related to oncogenic pathways, tumor mutations, and immune function. According to the results of the enrichment analysis, ERscore may control the biological activity of tumors by taking part in a number of oncogenic hallmark pathways. Signaling pathways such as E2F_TARGETS, MYC_TARGETS_V1, G2M_CHECKPOINT, ANDROGEN_RESPONSE are closely related to patient prognosis. Upregulation of E2F signaling has been reported to promote PRAD proliferation and progression [30]. Aberrant activation of the MYC pathway can switch PRAD cells to a “dormant mode” to escape attack from the immune system and antitumor therapy [31]. Activation of the ANDROGEN_RESPONSE pathway can independently promote PRAD progression, and its therapeutic targeting is a well-known treatment for PRAD [32].

Later, we explored the genetic mutations between the high- and low-risk groups. Mutant tp53 was found to activate UPR regulator ATF6 and suppress pro-apoptotic factors JNK and CHOP, thereby enhancing tumor cell resistance to ERS [33, 34]. We speculated that upregulated TP53 mutations in the high-risk group might suppress ERS in PRAD, resulting in worse prognostic outcomes. Further analysis uncovered increased mutations of MYC, NRF, and PI3K pathways in the high-risk group. Notably, N-Myc was reported to promote the malignant progression of PRAD by regulating FSCN1 [35]. In addition, N-Myc differentially modulated miR-421/ATM complex to motivate androgen deprivation therapy and Enzalutamide resistance in PRAD [36]. Combined with the results of GSVA and GSEA, we believed that ERscore was closely related to the mutation and abnormal activation of the MYC pathway. Moreover, the PI3K signaling cascade is one of the most frequently upregulated pathways in PRAD. It can promote tumor growth and therapeutic resistance to current treatment options by enhancing multiple downstream signaling events [37]. In addition, we found that ERscore and TMB were positively correlated, and the combination of ERscore and TMB can better predict the patient prognosis. Since TMB reflects a mutagenesis process induced by environmental and intracellular factors, it has become a useful biomarker in certain cancer types to identify patients who will benefit from immunotherapy [38, 39]. Our results suggest that various mutational anomalies in tumor genes and pathways that control PRAD development and progression may be caused by ERscore.

We found that patients in the high-risk group had higher levels of immune cell infiltration and expression of immunosuppressive receptors and immunosuppressive ligands. Therefore, we supposed that these genes may accelerate immune tolerance in PRAD, thereby compromising the patient’s BCRF survival. No significant differences were found in predicting response to immunotherapy among patients in the high- and low-risk groups. Given that immunotherapy is not effective in PRAD, chemotherapy response was examined in PRAD patients with various ERscores. The results showed that 5 Fluorouracil, Cyclopamine, Imatinib, and Salubrinal had a better effect in the high-risk group. It was reported that 5 Fluorouracil combined with radiation therapy can be used to treat locally advanced PRAD [40]. In addition, Cyclopamine was demonstrated to block the hedgehog signaling pathway, leading to long-term regression of PRAD without recurrence [41, 42]. Interestingly, the combined administration of Salubrinal and Bortezomib inhibited the ER-related protein degradation pathway and may serve as a therapeutic option for PRAD with serine protease overexpression [43]. Our study highlighted the involvement of ERscore in multiple immune responses and found that chemotherapy may be more effective than immunotherapy in high-risk patients.

There are extensive interconnections between malignant and stromal cells in TME, including early tumor recruitment and activation of a primitive precancerous stroma composed of stromal cells. Stromal cells, in turn, promote phenotypic changes in nearby tumor cells, which in turn signal stromal cells to continue their reprogramming [44]. Therefore, we investigated the cell-to-cell communications in the PRAD TME with the scRNA-seq data. Increased communication was found in the high ERS score group, suggesting the ERS-based signature played an important role in intercellular communication. Further analysis revealed that EGF, VEGF, PDGF, and IGF signaling pathways were strengthened in the high ERS score group compared to the low ERS score group. Notably, the EGF signaling was reported to be a vital upstream of AKT/δ-catenin/p21 for motivating PADR proliferation and metastasis [45]. In addition, it was demonstrated that the VEGF pathway can be activated by androgen and thereby promote PRAD angiogenesis and progression [46]. Generally, patients in the high and low ERS score groups showed differences in intercellular communication, and ERS-based signature may be involved in various oncogenic pathways to regulate PRAD growth and development.

Among the vital genes that make up the ERscore, ASNS was strongly associated with PRAD prognosis. It was reported that the activation of ASNS transcription and translation can deprotect cell survival to promote tumorigenesis [47, 48]. Meanwhile, the downregulation of ASNS can inhibit gastric cancer cell proliferation and reduce the colony formation ability of tumor cells [49]. In melanoma and breast cancer cells, downregulation of ASNS induced cell cycle arrest, which significantly inhibited the growth of cancer cells [50, 51]. In our research, *in vitro* experiments demonstrated that the knockdown of ASNS significantly inhibited the proliferation, migration and invasion of PRAD cells. The above findings suggested an oncogenic role of ASNS in PRAD, and knockdown of ASNS may be a potential therapeutic option.

This study comprehensively analyzed high-throughput sequencing data from multiple databases, constructed the ERscore, and validated it by independent cohorts. Our study also has certain limitations. Because this study is retrospective, data on the effects of treatment and relapse in PRAD patients are needed to validate our conjectures. Even if experimental validation is performed in ASNS, further *in vivo* or *in vitro* tests will be collected to investigate the gene’s specific processes in greater depth.

## CONCLUSIONS

A novel ERS-related signature constructed in this study can effectively predict the BCRF survival probability of PRAD patients. In addition, this study provides new insights into the mechanisms of PRAD development and provides potential therapeutic markers for PRAD patients.

## Supplementary Materials

Supplementary Figures

Supplementary Table 1
