# Supplementary material for: An endoplasmic reticulum stress-related signature featuring ASNS for predicting prognosis and immune landscape in prostate cancer
Source: Aging (Albany NY). 2024 Jan 10;16(1):43–65. doi: 10.18632/aging.205280 (PMC10817364; doi:10.18632/aging.205280)
Supplement: Supplementary Figures [file aging-16-205280-s001.pdf]

SUPPLEMENTARY FIGURES

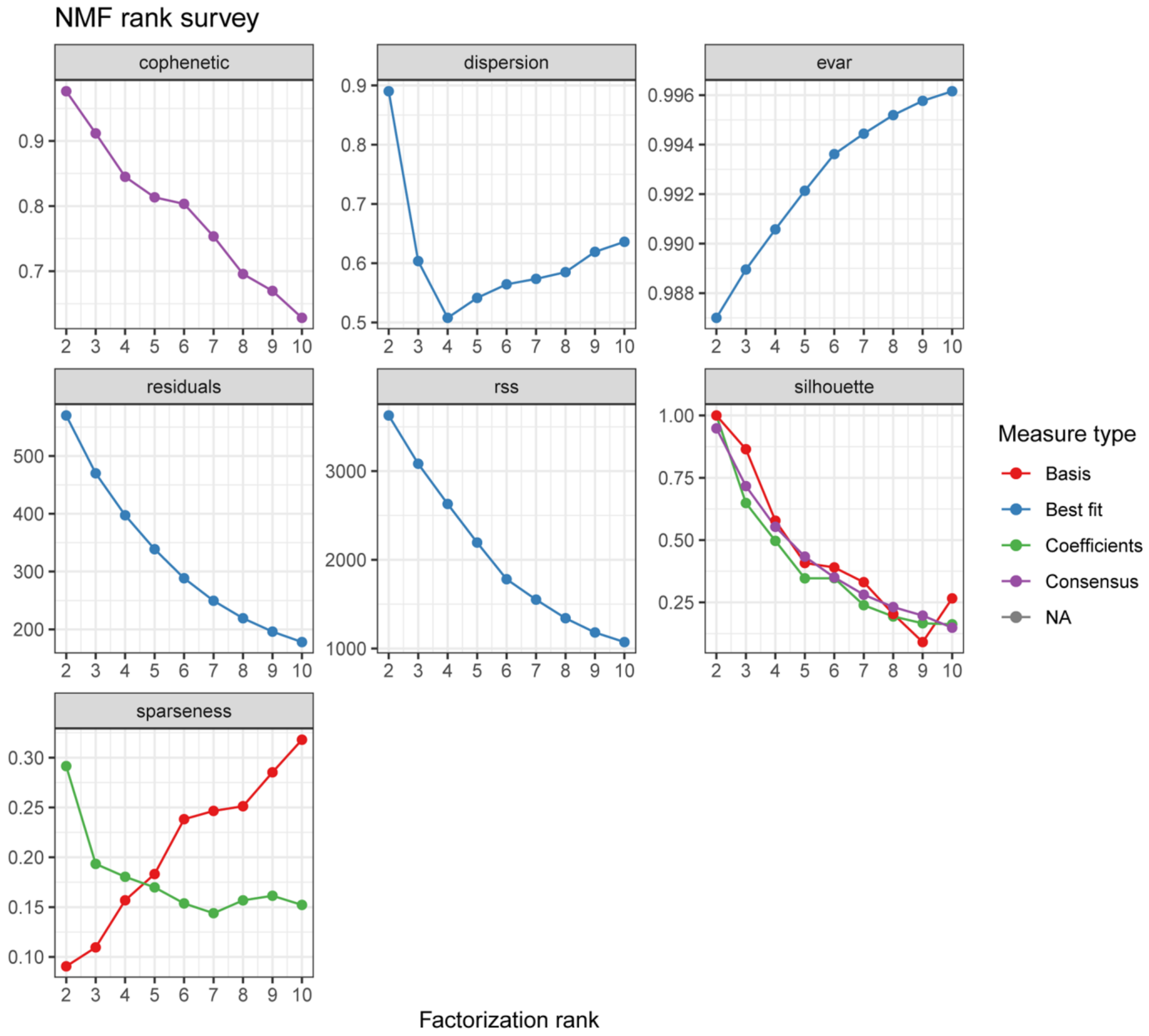

Supplementary Figure 1. The plots of cophenetic correlation coefficient reflected the stability of the cluster generated from NMF.

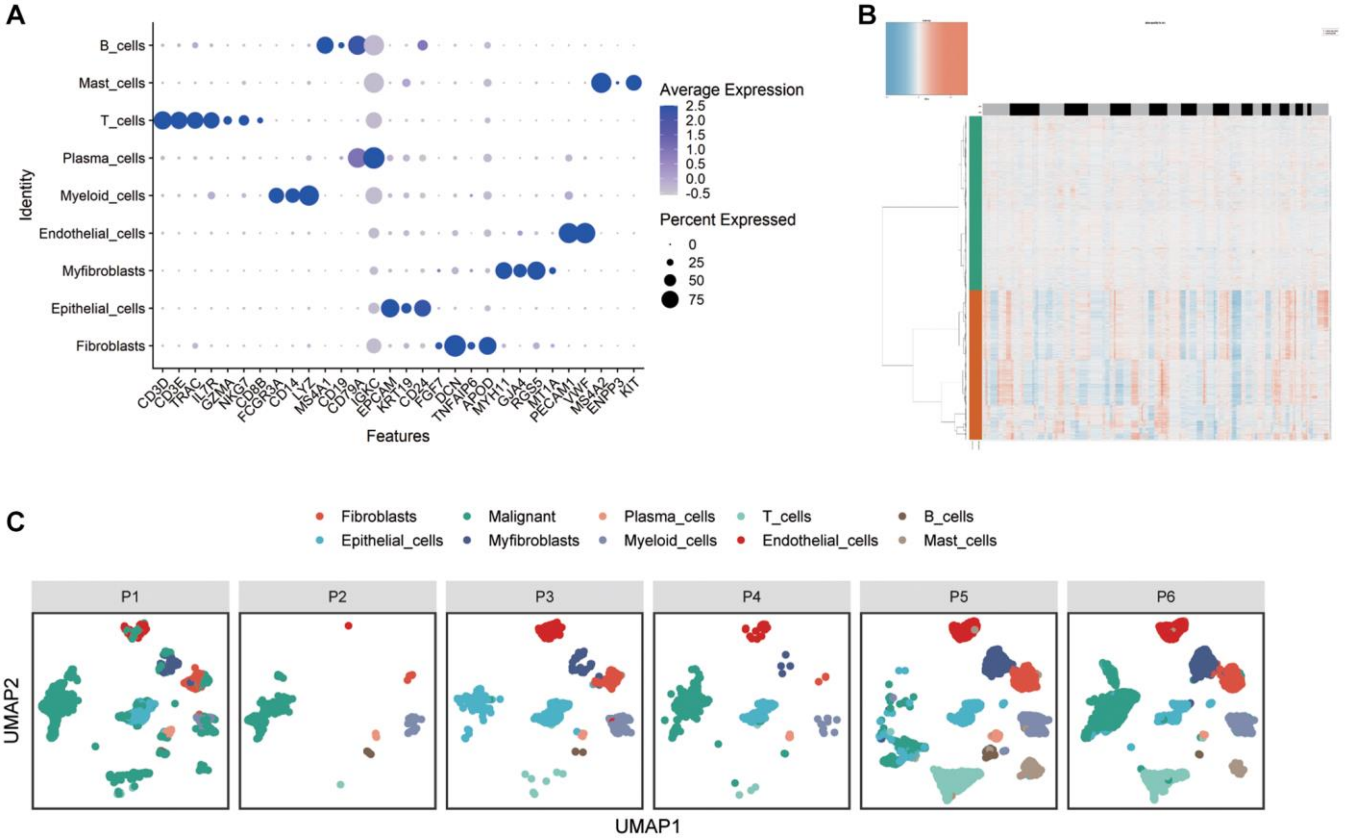

Supplementary Figure 2. Sample preprocessing of the scRNA-seq. (**A**) Markers of the main clusters. (**B**) The "copykat" R package was utilized to identify malignant cells. (**C**) UMAP plot revealed the composition of 10 main clusters from 6 patients.

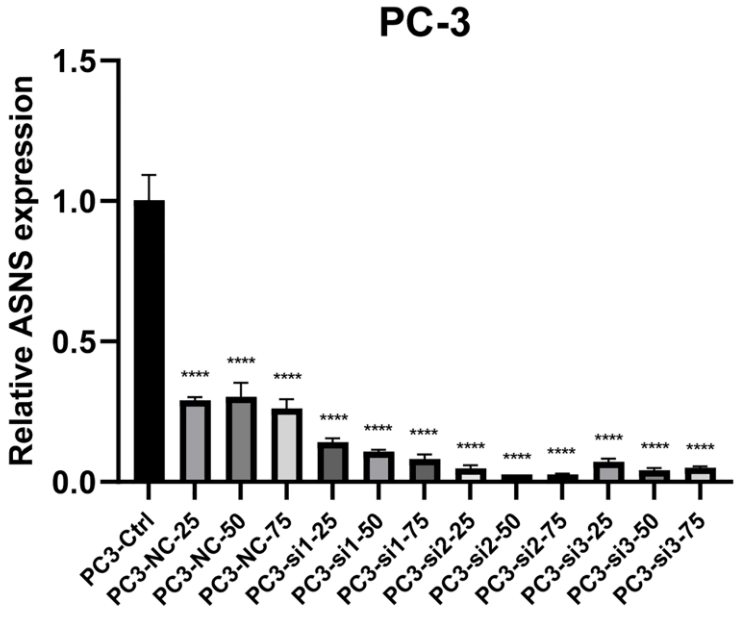

Supplementary Figure 3. Relative RNA expression for knockdown of ASNS. $^{****}p < 0.0001$.
