# Supplementary material for: An endoplasmic reticulum stress-related signature featuring ASNS for predicting prognosis and immune landscape in prostate cancer
Source: Aging (Albany NY). 2024 Jan 10;16(1):43–65. doi: 10.18632/aging.205280 (PMC10817364; doi:10.18632/aging.205280)
Supplement: Supplementary Table 1 [file aging-16-205280-s002.pdf]

SUPPLEMENTARY TABLE

Supplementary Table 1. Summary of ERS-related genes.

ERS-related gene			
ACADVL	DNAJB14	MBTPS1	SSR1
ADD1	DNAJB2	MBTPS2	STC2
AGR2	DNAJB9	NCK1	STT3B
AIFM1	DNAJC10	NCK2	STUB1
ALOX15	DNAJC18	NFE2L1	SULT1A3
AMFR	DNAJC3	NFE2L2	SVIP
ANKS4B	EDEM1	NPLOC4	SYVN1
ANKZF1	EDEM2	NRBF2	TARDBP
APAF1	EDEM3	OPA1	TATDN2
ARFGAP1	EEF2	OS9	TBL2
ASNS	EIF2AK2	P4HB	THBS1
ATF3	EIF2AK3	PARK7	THBS4
ATF4	EIF2AK4	PARP16	TLN1
ATF6	EIF2B5	PDIA2	TMBIM6
ATF6B	EIF2S1	PDIA3	TMCO1
ATG10	EP300	PDIA4	TMEM117
ATP2A1	ERLEC1	PDIA5	TMEM129
ATP2A2	ERLIN1	PDIA6	TMEM33
ATP6V0D1	ERLIN2	PDX1	TMEM67
ATXN3	ERN1	PIK3R1	TMTC3
AUP1	ERN2	PIK3R2	TMUB1
BAK1	ERP27	PLA2G6	TMUB2
BAX	ERP29	PMAIP1	TMX1
BBC3	ERP44	PML	TNFRSF10B
BCAP31	EXTL1	POMT2	TOR1A
BCL2	EXTL2	PPP1R15A	TP53
BCL2L1	EXTL3	PPP1R15B	TPP1
BCL2L11	FAF2	PPP2CB	TRAF2
BFAR	FBXO2	PPP2R5B	TRIB3
BHLHA15	FBXO6	PREB	TRIM13
BOK	FCGR2B	PSMC6	TRIM25
BRSK2	FGF21	PTPN1	TSPYL2
CALR	FICD	PTPN2	TTC23L
CASP4	FKBP14	RASGRF1	TXNDC12
CAV1	FLOT1	RASGRF2	UBA5
CCDC47	FOXRED2	RCN3	UBAC2
CCL2	GET4	RHBDD1	UBE2G2
CCND1	GFPT1	RNF103	UBE2J1

CDK5RAP3	GORASP2	RNF121	UBE2J2
CEBPB	GOSR2	RNF139	UBE2K
CFTR	GRINA	RNF175	UBE4A
CHAC1	GSK3A	RNF183	UBE4B
CLU	GSK3B	RNF185	UBQLN1
COPS5	HDGF	RNF186	UBQLN2
CREB3	HERPUD1	RNF5	UBXN1
CREB3L1	HM13	RNFT1	UBXN4
CREB3L2	HSP90B1	SCAMP5	UBXN6
CREB3L3	HSPA13	SDF2	UBXN8
CREB3L4	HSPA1A	SDF2L1	UFC1
CTDSP2	HSPA5	SEC16A	UFM1
CTH	HYOU1	SEC31A	UGGT1
CUL7	IGFBP1	SEC61B	UGGT2
CXXC1	ITPR1	SEL1L	USP13
DAB2IP	JKAMP	SERINC3	USP14
DCTN1	JUN	SERP1	USP19
DDIT3	KDELR3	SERP2	USP25
DDRGK1	KLHDC3	SESN2	VAPB
DDX11	LMNA	SGTA	VCP
DERL1	LRRK2	SGTB	WFS1
DERL2	MAGEA3	SHC1	WIPI1
DERL3	MAN1B1	SIRT1	YIF1A
DNAJB11	MANF	SRPRB	YOD1
DNAJB12	MAP3K5	SRPX	ZBTB17
